# Nuclear Kaiso Expression Is Associated with High Grade and Triple-Negative Invasive Breast Cancer

**DOI:** 10.1371/journal.pone.0037864

**Published:** 2012-05-25

**Authors:** Jeroen F. Vermeulen, Robert A. H. van de Ven, Cigdem Ercan, Petra van der Groep, Elsken van der Wall, Peter Bult, Matthias Christgen, Ulrich Lehmann, Juliet Daniel, Paul J. van Diest, Patrick W. B. Derksen

**Affiliations:** 1 Department of Pathology, University Medical Center Utrecht, Utrecht, The Netherlands; 2 Division of Internal Medicine and Dermatology, University Medical Center Utrecht, Utrecht, The Netherlands; 3 Department of Pathology, Radboud University Nijmegen Medical Centre, Nijmegen, The Netherlands; 4 Institute of Pathology, Hannover Medical School, Hannover, Germany; 5 Department of Biology, McMaster University, Hamilton, Onatrio, Canada; Health Canada, Canada

## Abstract

Kaiso is a BTB/POZ transcription factor that is ubiquitously expressed in multiple cell types and functions as a transcriptional repressor and activator. Little is known about Kaiso expression and localization in breast cancer. Here, we have related pathological features and molecular subtypes to Kaiso expression in 477 cases of human invasive breast cancer. Nuclear Kaiso was predominantly found in invasive ductal carcinoma (IDC) (p = 0.007), while cytoplasmic Kaiso expression was linked to invasive lobular carcinoma (ILC) (p = 0.006). Although cytoplasmic Kaiso did not correlate to clinicopathological features, we found a significant correlation between nuclear Kaiso, high histological grade (p = 0.023), ERα negativity (p = 0.001), and the HER2-driven and basal/triple-negative breast cancers (p = 0.018). Interestingly, nuclear Kaiso was also abundant in *BRCA1*-associated breast cancer (p<0.001) and invasive breast cancer overexpressing EGFR (p = 0.019). We observed a correlation between nuclear Kaiso and membrane-localized E-cadherin and p120-catenin (p120) (p<0.01). In contrast, cytoplasmic p120 strongly correlated with loss of E-cadherin and low nuclear Kaiso (p = 0.005). We could confirm these findings in human ILC cells and cell lines derived from conditional mouse models of ILC. Moreover, we present functional data that substantiate a mechanism whereby E-cadherin controls p120-mediated relief of Kaiso-dependent gene repression. In conclusion, our data indicate that nuclear Kaiso is common in clinically aggressive ductal breast cancer, while cytoplasmic Kaiso and a p120-mediated relief of Kaiso-dependent transcriptional repression characterize ILC.

## Introduction

Kaiso was initially identified as a binding partner of the adherens junction (AJ) complex member p120-catenin (p120) [1]. Kaiso is a member of the BTB/POZ-ZF (Broad complex, Tramtrak, Bric á brac/Pox virus and zinc finger) family of transcription factors [2] consisting of approximately 60 BTB/POZ-ZF members that include the cancer-associated B cell lymphoma 6 (*BCL6*), lymphoma-related factor (*LRF*), and hypermethylated in cancer (*HIC1*) genes (reviewed in [3]). Kaiso (also known as zinc finger- and BTB domain-containing protein 33; ZBTB33) interacts with its target gene promoters via two distinct mechanisms: via sequence-specific Kaiso binding sites (KBS), consisting of the consensus sequence CTGCNA, or via methylated CpG dinucleotides [4]–[7]. Although Kaiso can act as a transcriptional activator [8], it mainly acts as a transcriptional repressor by binding to the promoters of its target genes. This interaction can be inhibited by p120 binding to a region flanking Kaiso’s ZF motifs [1] and results in expression of distinct target genes [9], [10]. Kaiso has been shown to directly repress canonical Wnt targets via TCF/LEF family members [11], [12]. These target genes include the matrix metalloprotease *Matrilysin* (*aka MMP7*), *CCND1*, *Siamois, Fos* and *Myc* [4], [12]. In addition, Kaiso can regulate expression of *Wnt11*, a regulator of directed cell movement and morphogenesis [10].

While there is data demonstrating a role for Kaiso in early vertebrate development [12], [13], data implicating Kaiso-mediated regulation of gene transcription in cancer are scarce. Kaiso expression and sub-cellular localization seems dynamic and highly dependent on tumor type and micro-environmental conditions [14], [15]. Interestingly, *Kaiso*-null mice show resistance to intestinal cancer characterized by a delayed onset of tumor development, decreased tumor size, and prolonged survival when crossed with *APC^MIN/+^* mice [16].

Loss of E-cadherin and subsequent disruption of AJ function is strongly linked to breast cancer development and progression (reviewed in [17]). Using tissue-specific and conditional mouse models, we have established a causal relationship between early inactivation of E-cadherin and formation of invasive lobular carcinoma (ILC) [18], [19]. While β-catenin is rapidly degraded upon loss of E-cadherin [20], [21], p120 translocates and resides in the cytosol [22], where it regulates anchorage-independent tumor growth and metastasis through Mrip-dependent activation of the Rock pathway [20]. In addition, cytoplasmic p120 has been implicated in the acquisition of motility and invasiveness in E-cadherin negative breast cancer [23], [24]. The structure of p120 reveals a number of domains including a protein-protein interaction Armadillo (Arm) domain consisting of 10 Armadillo repeats. This domain mediates not only the interaction with cadherins but also p120 binding to the transcriptional repressor Kaiso, probably in a mutually exclusive manner [1].

Given the importance of p120 in the pathobiology of breast cancer, and its regulation of Kaiso-mediated transcriptional repression, we performed a comprehensive analysis of Kaiso expression and localization to pathological features and molecular subtypes in 477 cases of invasive breast cancer.

## Materials and Methods

### Patients

The study population was derived from the archives of the Departments of Pathology of the University Medical Center Utrecht, Utrecht, and the Radboud University Nijmegen Medical Centre, Nijmegen, The Netherlands. These comprised 477 cases of invasive breast cancer, including cases with a *BRCA1* germ-line mutation as previously described [25]. Histological grade was assessed according to the Nottingham scheme, and mitotic activity index (MAI) was assessed as before [26]. From representative donor paraffin blocks of the primary tumors, tissue microarrays were constructed by transferring tissue cylinders of 0.6 mm (3 cylinders per tumor) from the tumor area, determined by a pathologist based on haematoxylin-eosin stained slides, using a tissue arrayer (Beecher Instruments, Sun Prairie, WI, USA) as described before [27]. The use of anonymous or coded leftover material for scientific purposes is part of the standard treatment contract with patients in The Netherlands [28]. Ethical approval was not required.

**Figure 1:**
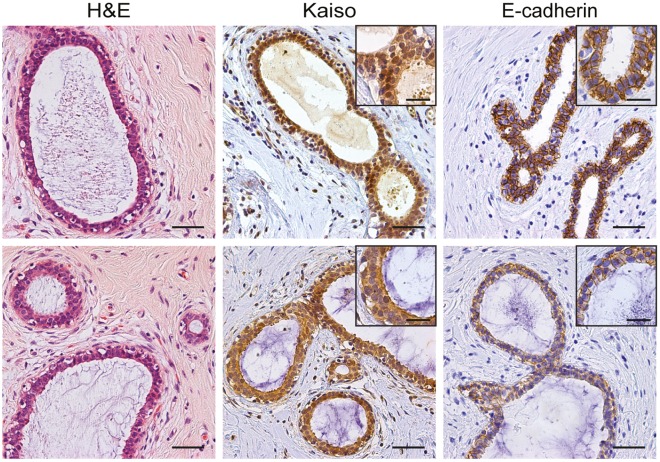
Kaiso expression in normal breast epithelium. Sections were stained using immunohistochemistry for Kaiso (middle panel) or E-cadherin (right panel). Kaiso expression in normal breast epithelium is heterogeneous; high nuclear expression (top row) versus only cytoplasmic expression (bottom row) is found with equal frequency. Left panels show a haematoxylin and eosin (H&E) staining. Size bars equal 50 µm, in inserts 25 µm.

### Immunohistochemistry

Immunohistochemistry was carried out on 4 µm thick sections. After deparaffination and rehydration, endogenous peroxidase activity was blocked for 15 min in a 46 mM citric acid-100 mM sodiumphosphate buffer solution pH5.8 containing 0.3% hydrogen peroxide. After antigen retrieval, i.e. boiling for 20 min in 10 mM citrate pH6.0 (Kaiso, p120, PR), Tris/EDTA pH9.0 (E-cadherin, ERα, HER2), or Prot K (0.15 mg/ml, DAKO, Glostrup Denmark) for 5 min at room temperature (EGFR), a cooling period of 30 min preceded the primary antibody incubation. Kaiso (clone 6F, Upstate, Billerica, MA, USA) [29] 1∶100; E-cadherin (clone 4A2C7, Zymed, Invitrogen, Breda, The Netherlands) 1∶200; ERα (clone ID5, DAKO) 1∶80; PR (clone PgR636, DAKO) 1∶25; HER2 (SP3, Neomarkers, Duiven, The Netherlands) 1∶100 were diluted in PBS containing 1% BSA and incubated for 1 h at room temperature. Primary antibodies against p120 (cat 610134, BD Transduction Labs, San Diego, CA, USA) 1∶500 and EGFR (clone 31G7, Zymed, Invitrogen) 1∶30 were diluted in PBS containing 1% BSA and incubated over night at 4°C. The signal was amplified using Powervision poly-HRP anti-mouse, rabbit, rat (DPVO-HRP, Immunologic, Duiven, The Netherlands) or the Novolink kit (Leica, Rijswijk, The Netherlands) (in the case of EGFR) and developed with diaminobenzidine, followed by counterstaining with haematoxylin, dehydration in alcohol, and mounting. Appropriate negative and positive controls were used throughout.

### Scoring of Immunohistochemistry

All scoring was done blinded to patient characteristics and results of other staining by two independent observers. E-cadherin and EGFR stainings were scored using the DAKO/HER2 scoring system for membranous staining. Membranous scores 1+, 2+, and 3+ were considered positive, except for HER2 where only a score of 3+ was considered positive. Kaiso staining was scored based on localization and by counting the positive tumor nuclei, considering samples with more than 5% positive tumor nuclei as positive. Using thresholds of 1 or 10% for scoring nuclear accumulation as positive did not change the results. p120 staining was scored based on the localization as membranous or cytoplasmic.

Based on ERα, PR, and HER2 immunohistochemistry, tumors were classified as luminal (ERα and/or PR positive), HER2-driven (ERα-, PR-, HER2+), or basal-like/triple negative (ERα-, PR-, HER2- with or without EGFR expression), the immunohistochemical surrogate [30] of the original Sorlie/Perou classification [31].

### Statistics

Statistical analysis was performed using IBM SPSS Statistics version 18.0 (SPSS Inc., Chicago, IL, USA). Associations between categorical variables were examined using the Pearson’s Chi-square test and associations between continuous variables using the Student’s T-test. P-values <0.05 were considered to be statistically significant.

### Cell Culture

Origin and culture of the mouse cell lines Trp53^Δ/Δ^-3, Trp53^Δ/Δ^-4 and mILC-1 were described before [20]. ILC cell line IPH-926 was cultured as described [32]. Human breast cancer cell line MCF10a was obtained from ATCC (validated by STR profiling), and cultured in DMEM/F12 (Invitrogen, Breda, the Netherlands) supplemented with 10 mg/L insulin, 20 µg/L EGF, 100 µg/L cholera toxin, and 500 µg/L hydrocortisone (Sigma, Zwijndrecht, The Netherlands). All media contained 10% fetal calf serum, 100 IU/ml penicillin, and 100 µg/ml streptomycin. All cell lines were maintained at 37°C in a 5% CO_2_ humidified atmosphere.

**Figure 2:**
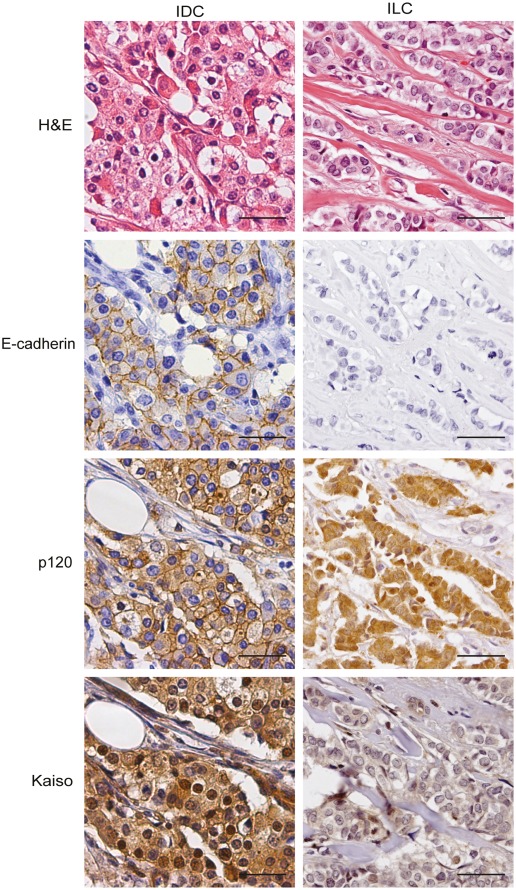
E-cadherin and p120 membrane localization correlates with nuclear Kaiso expression. IDC (left panels) and ILC (right panels) were stained for E-cadherin, p120, and Kaiso using immunohistochemistry. Note the association between membrane-localized E-cadherin and p120, and high nuclear Kaiso in IDC. In contrast, ILC is characterized by loss of E-cadherin, and expression of cytoplasmic p120, which correlates with absence of nuclear Kaiso. Size bars equal 50 µm.

**Table 1:** Clinicopathological characteristics of 477 invasive breast cancer patients studied for the expression of Kaiso.

Feature	Grouping	N or value	%
Age (years)	Mean	60	
	Range	28 to 88	
Histological type	IDC	312	65.4
	ILC	130	27.3
	Other	35	7.3
Tumor size (cm)	≤2	207	43.4
	>2 and ≤5	213	44.7
	>5	50	10.5
	Not available	7	1.4
Histological grade	1	85	17.8
	2	165	34.6
	3	208	43.6
	Not available	19	4.0
MAI #	≤12	241	50.5
	≥13	236	49.5
Lymph node status	Negative*	229	48.0
	Positive**	223	46.8
	Not available	25	5.2

# per 2 mm^2^.

* negative  =  N0 or N0(i+).

** positive  =  ≥N1mi (according to TNM 7^th^ edition, 2010).

**Table 2:** Correlation of cytoplasmic Kaiso with clinicopathological features in invasive breast cancer.

		Cytoplasmic Kaiso expression		
Feature	N	Negative	Positive	p-value
		N (%)	N (%)	
Histological type				
IDC	312	45 (14.4)	267 (85.6)	
ILC	130	5 (3.8)	125 (96.2)	
Other	35	5 (14.3)	30 (85.7)	**0.006**
Histological grade				
1	85	12 (14.1)	73 (85.9)	
2	163	16 (9.8)	147 (90.2)	
3	207	25 (12.1)	182 (87.9)	0.585
Tumor size (cm)				
≤2	205	22 (10.7)	183 (89.3)	
>2 and ≤5	213	29 (13.6)	184 (86.4)	
>5	49	3 (6.1)	46 (93.9)	0.296
MAI (per 2 mm^2^)				
≤12	238	29 (12.2)	209 (87.8)	
≥13	236	26 (11.0)	210 (89.0)	0.691
Lymph node status				
Negative	229	28(12.2)	201(87.8)	
Positive	223	25 (11.2)	198 (88.8)	0.737

**Table 3:** Correlation of nuclear Kaiso with clinicopathological features in invasive breast cancer.

		Nuclear Kaiso expression		
Feature	N	Low (<5%)	High (≥5%)	p-value
		N (%)	N (%)	
Histological type				
IDC	312	211 (67.6)	101 (32.4)	
ILC	130	107 (82.3)	23 (17.7)	
Other	35	25 (71.4)	10 (28.6)	**0.007**
Histological grade				
1	85	67 (78.8)	18 (21.2)	
2	165	125 (75.8)	40 (24.2)	
3	208	136 (65.4)	72 (34.6)	**0.023**
Tumor size (cm)				
≤2	207	154 (74.4)	53 (25.6)	
>2 and ≤5	213	149 (70.0)	64 (30.0)	
>5	50	37 (74.0)	13 (26.0)	0.573
MAI (per 2 mm^2^)				
≤12	241	188 (78.0)	53 (22.0)	
≥13	236	155 (65.7)	81 (34.3)	**0.003**
Lymph node status				
Negative	229	168 (73.4)	61(26.6)	
Positive	223	158 (70.9)	65 (29.1)	0.552

### Immunofluorescence

Cells were cultured on coverslips and fixed in methanol for 10 minutes, permeabilized using 0.3% Triton-X100/PBS and subsequently blocked using 4% BSA (Roche, Woerden, The Netherlands). Cover slips were incubated with mouse anti Kaiso 1∶500 (clone 6F) in 4% BSA for 1 hour at room temperature. Subsequently, cells were incubated in 4% BSA with goat-anti-mouse Alexa 488 (1∶600; Molecular Probes, Breda, The Netherlands) for 1 hour. Next, cells were incubated with TRITC-conjugated mouse anti-p120 1∶300 (clone 98/pp120, BD Biosciences) overnight at 4°C. Cover slips were mounted using Vectashield mounting medium (Vector Laboratories, Burlingame, CA, USA). Samples were analyzed by confocal laser microscopy.

### Luciferase Reporter Assay

Cells were cultured in 6-wells culture plates and grown to 40–50% confluency. Next, cells were transfected with either 600 ng of the Kaiso-specific reporter (pGL3-4XKBS), a mutated Kaiso reporter (pGL3-4XKBS CAmut) or empty vector (pGL3-Control) and co-transfected with 5 ng Renilla (pRL-CMV, Promega, Leiden, The Netherlands) for normalization of transfection efficiency, using Lipofectamine 2000 (Invitrogen) according to manufactures instructions. In addition, cells were transfected with 400 ng effector plasmid consisting either of pC2-p120 isoform 1a, pcDNA3.1-Kaiso or empty vector (pcDNA3.1). The transfection mixture was added to the cells and incubated for 2.5 hours. Followed by replacement of the transfection mixture by complete medium.

Two days post-transfection cells were washed twice with PBS, lysed by scraping in 200µl Passive Lysis Buffer (Promega) and subjected to a freeze-thaw cycle. Cellular debris was spun down at 5,000 g at 4°C for 5 minutes and supernatants were collected. Bioluminescence was measured in 50µl sample with Dual Luciferase Reporter assay system (Promega) on a Lumat LB9507 Luminometer (Berthold Technologies, Vilvoorde, Belgium) according manufactures instructions.

**Table 4:** Correlation of nuclear Kaiso with the molecular subtypes of breast cancer in invasive breast cancer.

		Nuclear Kaiso expression		
Feature	N	Low (<5%)	High (≥5%)	p-value
		N (%)	N (%)	
Perou/Sorlie classification				
Luminal	386	288 (74.6)	98 (25.4)	
HER2-driven	19	13 (68.4)	6 (31.6)	
Basal/Triple Negative	72	42 (58.3)	30 (42.7)	**0.018**
ERα				
Positive	378	285(76.0)	93 (24.0)	
Negative	99	58 (58.6)	41 (41.4)	**0.001**
PR				
Positive	276	206 (74.6)	70 (25.4)	
Negative	199	135 (67.8)	64 (32.2)	0.104
HER2				
Positive	45	29 (64.4)	16 (35.6)	
Negative	431	313 (72.6)	118 (27.4)	0.246
BRCA1				
Mutation carrier	21	6 (28.6)	15 (71.4)	
Sporadic	324	229 (70.7)	95 (29.3)	**0.001**
EGFR				
Positive	80	49 (61.3)	31 (38.7)	
Negative	395	293 (74.2)	102 (25.8)	**0.019**

## Results

### Kaiso Expression in Normal Breast Epithelium and Invasive Breast Cancer

In normal breast tissue, localization of Kaiso was observed in the cytosol of both luminal and myoepithelial cells (Figure 1). We detected nuclear Kaiso expression mainly in the luminal epithelial cells, which was heterogeneous while the number of cells showing nuclear Kaiso varied between ductal structures (5–35% of the cells)(Figure 1).

Next, we set out to analyze Kaiso expression in invasive breast cancer. We used a study population comprised of 312 (65.4%) IDC, 130 (27.3%) ILC, and 35 (7.3%) invasive breast cancer cases with other histology (Table 1). First, we scored absence or presence of cytoplasmic expression of Kaiso, since this variable has recently been linked to poor prognosis in non-small cell lung cancer (NSCLC) [33]. Although cytoplasmic expression of Kaiso was significantly different between the histological sub-types of breast cancer (p = 0.006), it was not associated with other clinicopathological features (Table 2 and Figure 2). Given that Kaiso functions as a transcriptional repressor, we scored nuclear expression in our breast cancer cohort. Since using thresholds of 1%, 5% or 10% for scoring of nuclear accumulation resulted in identical outcome and statistical significance (data no shown), we used 5% nuclear localization as a positive cut-off percentage. IDC expressed nuclear Kaiso more often than ILC (p = 0.007; Table 3; Figure 2), while exclusive cytoplasmic expression of Kaiso was a common feature of ILC. In addition, no significant difference was found between classical and pleomorphic lobular cancers (p = 0.237) (data not shown). For the other clinicopathological features, we observed that high-grade tumors and cancers with a MAI ≥13, had significantly more nuclear Kaiso than low-grade tumors (p = 0.023 and p = 0.003, respectively), while no significant differences were found for lymph node status and tumor size (Table 3).

**Table 5:** Correlation between functional adherens junctions and nuclear Kaiso expression in invasive breast cancer.

		Nuclear Kaiso expression		
Feature	N	Low (<5%)	High (≥5%)	p-value
		N (%)	N (%)	
E-cadherin				
Positive	327	220 (67.3)	107 (32.7)	
Negative	121	97 (80.2)	24 (19.8)	**0.008**
p120				
Membranous	320	214(66.9)	106 (33.1)	
Cytoplasmic	139	114 (82.0)	25 (18.0)	**0.001**

### Nuclear Kaiso Expression and Molecular Subtypes of Breast Cancer

Since Kaiso is implicated in transcriptional repression of specific target genes, and our data indicated that nuclear Kaiso correlated with histology and grading in our invasive breast cancer cohort, we performed a cross-comparison between nuclear Kaiso expression and the molecular subtypes of breast cancer. Nuclear Kaiso was significantly enriched in the basal/triple negative and HER2-driven breast cancers than luminal-type breast cancers (p = 0.018; Table 4). While we did not find differences in nuclear Kaiso expression in the context of PR and HER2 (p = 0.104 and p = 0.246, respectively), an inverse correlation between nuclear Kaiso and ERα expression was detected (p = 0.001) (Table 4). Moreover, *BRCA1*-associated breast cancers showed a significantly higher number of tumors expressing nuclear-localized Kaiso than sporadic carcinomas (71,4% versus 29,3%, respectively; p<0.001, Table 4).

### Localization of Kaiso, EGFR and the Adherens Junction in Breast Cancer

Expression of EGFR has been linked to prognosis in basal/triple-negative breast cancer [34], [35]. Because EGFR partly co-localizes with the AJ [36], and EGF stimulation can modulate AJ function through phosphorylation of Src, p120 and PKCδ [37], [38], we determined whether EGFR expression correlated with levels of membranous E-cadherin and nuclear Kaiso. Indeed, a strong association between EGFR and E-cadherin (p<0.001) was observed, which coincided with a higher prevalence of nuclear Kaiso expression in EGFR-expressing breast cancers (p = 0.019; Table 4).

Kaiso was identified as a p120-binding partner in a yeast two-hybrid screen, using p120 as bait [1]. Since then, several studies indicated that p120 controls relief of Kaiso-mediated transcriptional repression through binding and shuttling from and to the cytosol [9], [10]. Interestingly, this feature can be antagonized by E-cadherin expression, a key determinant in the differential diagnosis between IDC and ILC [39]. While approximately 90% of ILC cases show loss of E-cadherin expression, the majority of IDC cases have retained E-cadherin on the membrane [40]–[42]. Our data indicated that IDC and ILC show significant differences in cytoplasmic and nuclear Kaiso localization (Figure 2, Tables 3 and 4). The presence of AJs, *i.e.* membranous localization of E-cadherin and p120, strongly correlated with high nuclear Kaiso (p = 0.008 and p = 0.001, respectively; Table 5). Moreover, nuclear Kaiso inversely correlated with cytoplasmic p120 (p = 0.005), thus supporting the notion that loss of E-cadherin and subsequent translocation of p120 to the cytosol may control Kaiso localization.

**Figure 3:**
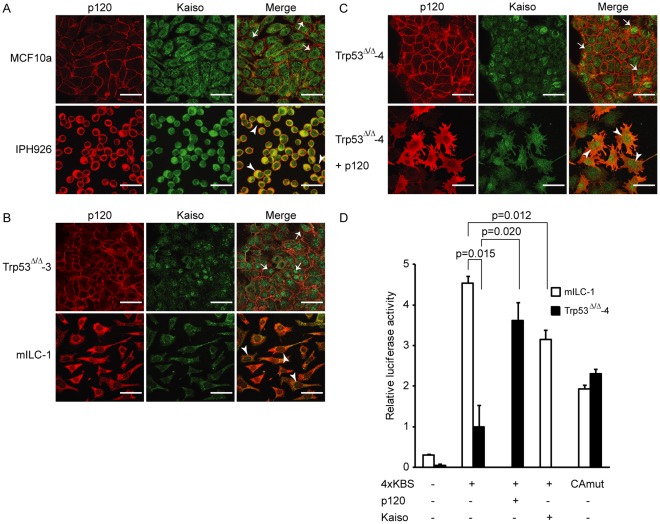
p120 and Kaiso localization in breast cancer cell lines. Human (A) and mouse (B) E-cadherin-expressing (top panels) and E-cadherin-deficient (bottom panels) breast cancer cell lines were stained for p120 (left panels) and Kaiso (middle panels). Right panels depict the merge of Kaiso (green) and p120 (red). Note the nuclear accumulation in MCF10a and Trp53^Δ/Δ^-3 (arrows; also upper panels in C) *versus* cytoplasmic Kaiso expression in human and mouse ILC (arrowheads; IPH-926 and mILC-1). (C) Overexpression of p120 in Trp53^Δ/Δ^-4 cells resulted in decreased nuclear accumulation of Kaiso (arrowheads; bottom panels) compared to untransfected Trp53^Δ/Δ^-4, which shows predominantly nuclear Kaiso (arrows; upper panels). Size bars equal 20 µm. (D) Kaiso-dependent reporter assay using the 4XKBS reporter in mILC-1 and Trp53^Δ/Δ^-4 cells. Upon overexpression of p120 in Trp53^Δ/Δ^-4 cells, Kaiso-dependent gene repression is attenuated, whereas exogenous expression of Kaiso in mILC-1 increased gene repression.

To substantiate these findings, we analyzed expression of Kaiso in MCF10a, a breast cancer cell line that expressed membranous E-cadherin and p120. Furthermore, since our data indicated that nuclear Kaiso and ILC were inversely correlated, we also used immunofluorescence to examine Kaiso localization in a recently generated and characterized bona fide human ILC cell line; IPH-926 [32]. Although Kaiso expression was observed in E-cadherin-expressing as well as in E-cadherin-mutant cells, nuclear Kaiso was enriched in MCF10a cells, whereas IPH-926 virtually lacked nuclear Kaiso (Figure 3A). In addition, we employed cell lines derived from conditional mouse models in which E-cadherin and/or p53 were somatically inactivated [19]. In agreement with our findings in human cell lines, we could detect nuclear Kaiso in an E-cadherin expressing and p53-deficient mammary carcinoma cell line (Trp53^Δ/Δ^-3), whereas mouse ILC (mILC) cells mainly lacked nuclear Kaiso (Figure 3B). Evidence that p120 could direct nuclear localization of Kaiso was obtained by overexpressing p120 in Trp53^Δ/Δ^-4 cells, which resulted in high cytoplasmic p120 and a reduction in nuclear Kaiso (Figure 3C). To determine the effect of p120 overexpression on Kaiso-dependent transcriptional repression, we performed reporter assays using a Kaiso reporter system (containing 4 tandem-repeats of the consensus Kaiso Binding Sequence; 4XKBS reporter). In line with our expression data, we observed that Kaiso-dependent transcriptional repression was significantly higher in Trp53^Δ/Δ^-4 than in mILC-1 cells (p = 0.015; Figure 3D). Furthermore, transcriptional repression of the 4XKBS reporter was attenuated by exogenous Kaiso expression in mILC-1 cells (Figure 3D). Finally, we observed that overexpression of p120 in Trp53^Δ/Δ^-4 cells resulted in decreased Kaiso-dependent transcriptional repression (Figure 3C and 3D), consistent with a decrease in nuclear Kaiso expression. These results support our findings in primary breast cancer samples and indicate that p120 controls localization of Kaiso and subsequent de-repression of Kaiso-dependent transcription in breast cancer.

## Discussion

In addition to the established role of BTB-POZ-ZF transcription factors in vertebrate development, increasing evidence emerges that these factors can function as oncogenes or tumor suppressors [43]. For instance, the BTB/POZ promyelocytic leukemia zinc finger (PLZF) has been identified as a translocation partner of the retinoic receptor alpha (RARα). In this setting, PLZF confers oncogenic potential through fusion to the hormone-binding domain of RARα, subsequent binding to its target sites and local recruitment of histone deacetylases [44]. Another well-studied BTB-POZ oncogene is BCL6, a protein that exerts its pro-tumorigenic functions by repression of target genes necessary for terminal B cell differentiation [45], [46]. In contrast, *HIC1* is a candidate tumor suppressor that is often found mutated or hypermethylated in human cancer [47]. However, unlike PLZF, BCL-6 and HIC1, it remains unclear whether Kaiso mislocalization or absence could drive malignancy.

Kaiso could function as an oncogene or as a tumor suppressor as it has been implicated in both transcriptional activation and repression [4], [8], [13]. In colon cancer, Kaiso may regulate methylation-dependent inhibition of tumor suppressors such as *CDKN2A* by binding to its methylated promoter. As a consequence, tumor cells are resistant to cell cycle arrest and chemotherapy-mediated cell death [48]. Interestingly, genetic Kaiso ablation results in a delay in intestinal tumorigenesis in the context of *APC* ^MIN/+^ mice [16], which suggests that Kaiso may indeed contribute to intestinal tumor progression through silencing of tumor suppressors. Conversely, Kaiso has been strongly implicated in regulation of Wnt signaling-related target genes [4], [11], [12], [49], [50]. Given its bi-modal nature of β-catenin-dependent regulation of Wnt signaling [51] and the overlap between TCF/LEF regulated genes and Kaiso targets, the effects of Kaiso on tumor development may be highly dependent on cell type and their dependency on (canonical) Wnt signals. In lung cancer, cytoplasmic Kaiso was correlated with poor prognosis [33]. Here it was proposed that the invasive phenotype of NSCLC might be regulated by nuclear export of Kaiso, which was mediated by phosphorylation of p120 isoform 3 [52]. Lung and other epithelial tissue differ substantially from breast with respect to cadherin expression and p120 function. For instance, condition p120 knockout in the skin, gastro-intestinal tract or oral cavity is tolerated and induces hyperplasia or tumor formation [53]–[56]. In contrast, p120 knock-out in the mammary gland is not tolerated and leads to apoptosis and subsequent cell clearance (our unpublished results), indicating that p120 family members may play tissue-specific redundant roles, as has been suggested for δ-catenin in non-small-cell lung cancer [57].

In this study we have performed to the best of our knowledge, the first comprehensive analysis of Kaiso expression in breast cancer, using a tissue micro array (TMA)-based collection of 477 invasive breast cancer cases. Previous studies had already indicated that localization of Kaiso may be highly variable depending on tumor type and environmental context [14]. Our data indicate that nuclear Kaiso expression correlates with the pathological and phenotypical traits of specific breast cancer sub-types that are linked to poor prognosis, *i.e.* high-grade, and basal/triple-negative breast cancer. These tumors were also associated with high EGFR expression, which is associated with worse prognosis for basal/triple-negative breast cancers [34], [35]. Our observation that *BRCA1*-associated hereditary breast cancers often showed high nuclear Kaiso, is in line with the finding that nuclear Kaiso is in general associated with high grade, basal-like and EGFR positive breast cancers. Since our data does not indicate differential E-cadherin expression and localization between sporadic and BRCA1-related invasive breast cancer, this cannot explain the increase in nuclear Kaiso localization. Future research will have to determine if and how other (p120-unrelated) events such as promoter methylation of specific genes may recruit Kaiso to the nucleus and initiate subsequent epigenetic silencing in BRCA1-related invasive breast cancer. We have furthermore shown that nuclear Kaiso correlated with the presence of membrane-localized E-cadherin and p120, a finding that is in line with the reported regulation of Kaiso by p120 [58]. In this scenario, p120 relieves transcriptional repression by Kaiso and as such may control shuttling of the p120/Kaiso complex to the cytosol [9]. Because most IDC retain a membrane-localized E-cadherin/p120 complex, our data confirmed this concept by showing that nuclear Kaiso correlated with tumors expressing E-cadherin. Also, since the basal-like and ERα negative high-grade tumors mainly reside in the E-cadherin-expressing IDC cohort, it supports the notion that p120 may regulate Kaiso distribution in breast cancer. The mechanism of this needs to be further elucidated.

ILC is characterized by loss of the AJ complex through early mutational inactivation of E-cadherin and subsequent translocation of p120 to the cytosol. If p120 were a major factor controlling Kaiso distribution, one would expect that the absence of nuclear Kaiso associated with ILC. Our data indeed conforms to this hypothesis by showing that exclusive cytoplasmic Kaiso expression is strongly correlated with the lobular phenotype. Together, these findings suggest that genes may be differentially regulated in IDC versus ILC as a result of differential Kaiso localization. This notion may therefore partly explain the differences in expression profiles that have been reported when comparing IDC and ILC [59], [60].

Recent data have indicated that phosphorylation of p120 can increase its binding to Kaiso and induce inhibition of canonical Wnt signaling [11]. It is well established that ILC expresses cytoplasmic p120 and does not activate canonical Wnt signals [20], [21], [61]. Although it is unclear if this mechanism controls expression of Kaiso targets in ILC, it clearly emphasizes the possible ramifications of Kaiso and its regulation by p120 in breast cancer. Moreover, we have recently shown that cytoplasmic translocation of p120 controls ILC tumor growth and metastasis through Mrip-dependent regulation of Rock1 signaling, while IDC does not appear to be contingent on these signals for anchorage-independence [20]. We envisage that differential cadherin-catenin localization in IDC and ILC and the signals that emanate from p120 may not only explain the lobular phenotype, but probably also control regulation of transcriptional regulation and cellular biochemistry. Although Kaiso’s target genes in breast cancer are unknown, our findings suggest that Kaiso may function as an oncogene in IDC through inhibition of tumor suppressor gene expression whereas in ILC, Kaiso might harbor tumor suppressor functions by p120-mediated relieve of transcriptional repression of oncogenic target genes. As such, it may have significant impact on the development of personalized cancer care since it suggests that the main breast cancer types may depend on diametrical mechanisms for tumor progression.
